# Occupational health hazards in sewage and sanitary workers

**DOI:** 10.4103/0019-5278.44691

**Published:** 2008-12

**Authors:** Rajnarayan R. Tiwari

**Affiliations:** Occupational Medicine Division, National Institute of Occupational Health, Ahmedabad, India

**Keywords:** Sanitary workers, sewage workers

## Abstract

An estimated 1.2 million scavengers in the country are involved in the sanitation of our surroundings. The working conditions of these sanitary workers have remained virtually unchanged for over a century. Apart from the social atrocities that these workers face, they are exposed to certain health problems by virtue of their occupation. These health hazards include exposure to harmful gases such as methane and hydrogen sulfide, cardiovascular degeneration, musculoskeletal disorders like osteoarthritic changes and intervertebral disc herniation, infections like hepatitis, leptospirosis and helicobacter, skin problems, respiratory system problems and altered pulmonary function parameters. This can be prevented through engineering, medical and legislative measures. While the engineering measures will help in protecting against exposures, the medical measures will help in early detection of the effects of these exposures. This can be partly achieved by developing an effective occupational health service for this group of workers. Also, regular awareness programs should be conducted to impart education regarding safer work procedures and use of personal protective devices.

## INTRODUCTION

Nearly a century after Mahatma Gandhi first called for the abolition of manual scavenging, the degrading practice continues. Between 2002 and 2003, the Indian Ministry for Social Justice and Empowerment admitted to the existence of 676,000 scavengers.[1] However, these figures may have been underestimated because scavenging is illegal. According to one survey by Bezwada Wilson of the Safai Karmachari Association, an estimated 12 lakh (1.2 million) scavengers are present in the country.[2] According to Sulabh, four to five million people were working as scavengers in 2005 and were often employed by the local civil bodies to clean excrement in public places.[1] This situation persists despite the fact that the Employment of Manual Scavengers and Construction of Dry Latrines (Prohibition) Act,[3] 1993, is in enforcement, which provides for the prohibition of the employment of manual scavengers as well as construction or continuance of dry latrines and for the regulation of construction and maintenance of water-seal latrines for assuring the dignity of the individual, as enshrined in the Preamble to the Constitution.

The working conditions of the sanitary workers have remained virtually unchanged for over a century. Using only a stick broom and a small tin plate, the sanitary workers clear feces from public and private latrines onto baskets or other containers, which they then carry on their heads to dumping grounds and disposal sites. A few, however, are provided with wheelbarrows or carts by the municipal authorities.

Apart from the social atrocities that these workers face, they are also exposed to certain health problems by virtue of their occupation. These health hazards include exposure to harmful gases, cardiovascular degeneration, musculoskeletal disorders, infections, skin problems and respiratory system problems.

## EXPOSURE TO HARMFUL GASES

The workers are commonly exposed to gases like hydrogen disulfide, methane, ammonia and carbon monoxide. Watt *et al*.[4] studied 26 sewer workers exposed to smell and found that 53.8% developed sub-acute symptoms including sore throat, cough, chest tightness, breathlessness, thirst, sweating, irritability and loss of libido. Severity of symptoms seemed to be dose related. Richardson[5] studied exposure to hydrogen sulfide in 68 sewer workers and found that the FEV_1_/FVC values were lower in sewer workers who had a high H_2_S exposure.

### Harmful effects of hydrogen sulfide

Hydrogen sulfide is a flammable gas, which burns with a blue flame, giving rise to sulphur dioxide, a highly irritating gas with a characteristic odor. Mixtures of hydrogen sulfide and air in the explosive range may explode violently.

Even at low concentrations, hydrogen sulfide has an irritant action on the eyes and the respiratory tract. Intoxication may be hyperacute, acute, subacute or chronic. Hydrogen sulfide enters the body through the respiratory system and is rapidly oxidized to form compounds of low toxicity. There are no accumulation phenomena and elimination occurs through the intestine, urine and the expired air.

In cases of slight poisoning, following exposure from 10 to 500 ppm, a headache may last several hours, pain in the legs may be felt and, rarely, there may be loss of consciousness. In moderate poisoning (from 500 to 700 ppm), there will be loss of consciousness lasting a few minutes but with no respiratory difficulty. In cases of severe poisoning, the subject drops into a profound coma with dyspnoea, polypnoea and a slate-blue cyanosis until breathing restarts. Tachycardia and tonic–clonic spasms are seen.

Inhalation of massive quantities of hydrogen sulfide will rapidly produce anoxia resulting in death by asphyxia. Epileptiform convulsions may occur and the individual falls apparently unconscious and may die without moving again. This is a syndrome characteristic of hydrogen sulfide poisoning in sewer workers. However, in such cases, exposure is often due to a mixture of gases including methane, nitrogen, carbon dioxide and ammonia.

In sub-acute poisoning, the eyes are affected by palpebral edema, bulbar conjunctivitis and mucopurulent secretion with, perhaps, a reduction in visual acuity—all of these lesions usually being bilateral. This syndrome is known to sugar and sewer workers as “gas eye”.

## MUSCULOSKELETAL DISORDERS

Osteoarthritic changes and intervertebral disc herniation are the common spinal abnormalities reported in these workers.[6] Friedrich[7] studied 255 sewage workers to determine the prevalence of spinal troubles (i.e., neck, upper back and lower back pain [LBP]). He reported that the 12-month prevalence rates of neck, upper back and LBP were 52.4%, 54.8% and 72.8%, respectively. The prevalence of spinal troubles increased with age. Work disability during the preceding 12 months due to LBP was significantly positively associated with age, disability, weekly duration of stooping and lifting 5 years previously and higher abnormal illness–behavior scores (odds ratio between 1.26 and 0.94).

## INFECTIONS

The modes of exposure for the various infections are as follows:

- The most common way is by hand-to-mouth contact during eating, drinking and smoking, or by wiping the face with contaminated hands or gloves or by licking splashes from the skin.
- By skin contact, through cuts, scratches or penetrating wounds, i.e., from discarded hypodermic needles. Certain organisms can enter the body through the surfaces of the eyes, nose and mouth.
- By breathing them in as dust, aerosol or mist.

The infections commonly studied among this group of workers include leptospirosis, hepatitis and *Helicobacter pylori* infection.

### Leptospirosis

Leptospirosis is an important occupational disease affecting people coming in contact with animals and their discharges. The occurrence of infection in ones workplaces is linked to the environment to which the worker is exposed and the adaptability of the organism in that working environment. Rodents usually abound in underground sewers and are carriers of leptospira. The urine of rodents and other animals present in that area is likely to contaminate these sewers. Leptospira are excreted in the urine of the infected animals. Thus, sewer workers are at a potential risk of leptospirosis. Ambekar *et al*.[8] studied 78 sewer workers from five different municipal wards in Pune to determine the evidence of past infection with leptospira using a microagglutination test. The prevalence rate was found to be 16.6%. Evidence of leptospiral infection was found to be maximum in sewer workers in the areas of the city that were infested with rodents and stray animals. De Serres *et al*.[9] found that sewer workers had a greater prevalence of antibodies against leptospirosis than controls (12% vs. 2%, P = 0.003).

### Hepatitis

Hepatitis A (HA) virus is the most frequently occurring vaccine-preventable disease. Although generally self limiting, acute hepatitis A is associated with substantial morbidity and related economic burden. Few studies reported an increased HAV antibody titer among sewage workers[10–11] whereas other studies suggest that workers in the solid waste industry may only theoretically be at an increased risk of acquiring infectious diseases occupationally.[12–13] Even a systematic review carried out by Glas *et al*.[14] does not confirm an increased risk of clinical HA in workers exposed to sewage. Vaidya[15] reported that a significant rise in anti-hepatitis E virus positivity (*P* < 0.05) was recorded in sewage workers working for > 5 years. Another case report suggests that sewer workers may be at an increased risk of contracting hepatitis C.[16] Another study by Arvanitidou *et al*.[17] among employees of a sewage company confirms that only exposure to sewage was independently associated with positivity for hepatitis B virus (HBV) infection (*P* < 0.001). They recommended that workers exposed to sewage should therefore be considered for vaccination against HBV.

### Helicobacter pylori

An increased risk for gastric cancer among sewage workers has been described in several studies. During the last decade, the bacterium *Helicobacter pylori* has emerged as one important risk factor for gastric cancer and is now considered a class I carcinogen by the International Agency for Research on Cancer. Friis *et al*.[18] studied the prevalence of immunoglobulin G (IgG) antibodies against *H. pylori* in a group of 289 municipal workers. The prevalence of IgG antibodies against *H. pylori* among sewage workers did not differ from that of the referents. However, an increase in the prevalence of IgG antibodies against *H. pylori* with increasing age was observed.

Apart from these commonly studied infections, several other infections like intestinal parasitic infections, gastroenteritis and Pontiac fever are also described among the sewage workers.

## DERMATITIS

Mostly, the dermatitis is a non-infective one. It results from irritation to mineral oil and tar. An outbreak of cases of airborne irritant contact dermatitis has been reported among incinerator workers employed in a sewage treatment facility.[19]

## RESPIRATORY SYMPTOMS AND FUNCTION

Several studies have been carried out to study the respiratory function of sewage workers, with all of them reporting that respiratory symptoms are common[20–22] among this group of workers. The respiratory function studies also revealed abnormal respiratory functions in these workers. These symptoms may be due to exposure to endotoxins and airborne bacteria by way of bioaerosols. Zuskin *et al*. reported that the baseline ventilatory capacity was significantly decreased compared with the predicted values in sewage workers. In particular, the values for FEF_25__ -50_ were reduced,[23] suggesting obstructive changes in smaller airways. They mentioned that sewage workers are exposed to different occupational noxious agents, which may lead to the development of chronic lung function changes.[24]

Thus, to summarize, the sewage and sanitary workers suffer mainly from chemical and biological hazards. This can be prevented through engineering, medical and legislative measures. The engineering measure should focus on making the process more mechanistic. These workers should also be benefited from occupational health services, which should include pre-placement and periodic health monitoring. Further effective implementation of the Employment of Manual Scavengers and Construction of Dry Latrines (Prohibition) Act, 1993, will help in the abolition of manual scavenging. Also, regular awareness programs should be conducted to impart education regarding safer work procedures and use of personal protective devices.
